# Supplementary material for: Disruption of cross-feeding interactions by invading taxa can cause invasional meltdown in microbial communities
Source: Proc Biol Sci. 2020 May 13;287(1927):20192945. doi: 10.1098/rspb.2019.2945 (PMC7287355; doi:10.1098/rspb.2019.2945)
Supplement: Simulation Model Script [file rspb20192945supp1.rtf]

#!/usr/bin/env Rscriptargs = commandArgs(trailingOnly=TRUE)## args should be the mean competition value, the proportion of cross-feeding, and the number of model runs ## args can be manually entered to run fewer simulations, make figure, or run script locally #args <- c(.52, .1, 10)library(combinat)library(data.table)library(abind)library(dplyr)library(scales)## Create necessary functionsmin.of.n <- function(vec, min.n){  sort.vec <- sort(vec, decreasing = T)  min.sub <- min(sort.vec[1:min.n])  return(min.sub)}#Create function to find the number of matches in a vectormatches.in.vec <- function(var, vec){  matches <- sum(vec == var)  return(matches)}#Variable inputs based on command line argumentscomp.mean <- as.numeric(args[1])#print(comp.mean)comp.sd <- comp.mean * .3    direct.prop <- as.numeric(args[2])num.runs <- as.numeric(args[3])result.list <- list()## Fixed paramsmin.comp <- .1inv.comp <- .9input.rate <- 200num.taxa <- 20 num.all.taxa <- num.taxa + 2num.mets.all <- 8 mets.req <- 5inv.mets.req <- 5 mets.excreted <- 3flush <- .1time.steps <- 40000 #if the model doesn't equilibrate in this time, it is restartedinput.all.mets <- Fflow.strength <- 1over.step.half <- 0over.step.full <- 0over.step.count <- 0stoch.comp <- T#create a vector to record number of taxa coexistingnum.coex <- vector()#create a vector to hold equilibrium met poolseq.mets <- matrix(numeric(0), nrow = num.mets.all, ncol = num.runs)#Create a vector for sum of metabolitesmets.all <- vector()#Create a vector for metabolites before invasionmets.all.preinv <- vector()#create a vector to record number of taxa coexisting BEFORE invadernum.coex.preinv <- vector()#create a vector to hold equilibrium met pools BEFORE invadereq.mets.preinv <- matrix(numeric(0), nrow = num.mets.all, ncol = num.runs)#create a vector to look at whether invader persistsinv.persist <- vector()#create a vector to look at whether second invader persistssecond.inv.persist <- vector()#create a vector to look at whether second invader persists without first invadersecond.inv.control.persist <- vector()#make a vector to hold final abundances of taxa before invasionfinal.abuns.preinv <- matrix(numeric(0), nrow = num.all.taxa, ncol = num.runs)#make a vector to hold final abundances of taxa final.abuns <- matrix(numeric(0), nrow = num.all.taxa, ncol = num.runs)#make a vector to hold when first inv addedinv.add.time <- rep(0, times = num.runs)#make a vector to hold when second inv addedsecond.inv.add.time <- rep(0, times = num.runs)#Create vector for invader equilibrium abundanceinv.eq.abun <- vector()#Create vector for second control invader equilibrium abuninv.control.eq.abun <- vector()#create a vector for diversity of metabolitesmets.div <- vector()#create a vector for diversity of metabolites after invasionmets.div.post <- vector()#create a vector to hold the flows per metabolite flows.per <- vector()#create a vector for metabolite evennessmets.ev <- vector()#create vector for number of metabolites exchangednum.mets.trade <- vector()#create vector for number of flows per met after invasionflows.per.post <- vector()#create vector for change in diversity post invasiondiv.change <- vector()#create vector for change in free metabolties post invasionmets.change <- vector()#create vector for change in number of individuals post invasion inds.change <- vector()#create vector for change in number of flows per metaboliteflows.per.change <- vector()#create vector for change in mnumber of metabolites exchangedmets.trade.change <- vector()#create a vector to record number of flows per extra (above number mets in) taxon supported by crossfeedingflows.per.extra <- vector()#record average competition coefficients before invasionavg.comp.pre <- vector()#record average competition coefficients after invasionavg.comp.post.inv <- vector()#create vector for redundancy of metabolite flowredundancy <- vector()#create vector for redundancy of metabolite flow after invasionredundancy.post <- vector()#Create vector for flows per taxon before and after invasionflows.per.taxon <- vector()flows.per.taxon.post <- vector()#Create vectors for redundancy of limiting nutrientsredun.lim <- vector()redun.lim.post <- vector()##########################################################################################################################################################################################run <- 1while(run <= num.runs ){  reqs <- vector()  n.mets.for.taxa <- vector()    for(k in 1:length(mets.req)){    all.mets.k <- unique(permn(c(rep(1, mets.req[k]), c(rep(0, num.mets.all - mets.req[k])))))    which.mets.k <- sample(seq(1, length(all.mets.k)), round(num.taxa / length(mets.req) ), replace = F)        reqs.k <- matrix(unlist(all.mets.k[which.mets.k]), ncol = round(num.taxa / length(mets.req) ), byrow = F)        reqs <- cbind(reqs, reqs.k)    dim(reqs)          n.mets.for.taxa <- c(n.mets.for.taxa, rep(mets.req[k], round(num.taxa / length(mets.req))) )  }  n.mets.for.taxa <- c(n.mets.for.taxa, inv.mets.req, inv.mets.req)    #define invader reqs  all.inv.reqs <- unique(permn(c(rep(1, inv.mets.req), c(rep(0, num.mets.all - inv.mets.req)))))  all.inv.reqs.mat <- matrix(unlist(all.inv.reqs), nrow = num.mets.all , byrow = F)  M1 = setkey(data.table(t(reqs)))    M2 = setkey(data.table(t(all.inv.reqs.mat)))  shared <- na.omit(    M2[M1,which=TRUE]  )  shared <- as.vector(shared)  ifelse(length(shared) >= 1,          which.inv.reqs <- sample(seq(1, dim(M2)[1], 1)[-shared], 2),          which.inv.reqs <- sample(seq(1, dim(M2)[1], 1), 1) )  inv.reqs1 <- M2[which.inv.reqs[1], ]  inv.reqs2 <- M2[which.inv.reqs[2], ]    reqs <- cbind(reqs, t(inv.reqs1), t(inv.reqs2))    dim(reqs)      #mets.in <- unlist(sample(unique(permn(c(rep(1, max(mets.req) ), c(rep(0, num.mets.all - max(mets.req)))))), 1))  mets.in <- reqs[, sample(seq(1, dim(reqs)[2] - 2, 1), 1)]  if(input.all.mets) mets.in <- rep(1, times = num.mets.all)    all.ex.poss <- matrix(as.numeric(!reqs), ncol = num.all.taxa)  how.many.ones <- function(vec, ones){    which.ones <- which(vec == 1)    keep.ones <- sample(which.ones, ones)    vec.zero <- rep(0, length(vec))    vec.zero[keep.ones] <- 1    return(vec.zero)  }  ex <- apply(all.ex.poss, 2, how.many.ones, ones = mets.excreted)    #Make a matrix holding the donor, recipient, and met for each possible directed flow   donors <- vector()  recipients <- vector()  met.flow <- vector()  for(q in 1:num.mets.all){    for(w in 1:num.all.taxa){      if(ex[q, w] == 1){        num.req <- sum(reqs[q, ])        donors <- c(donors, rep(w, num.req))        recipients <- c(recipients, which(reqs[q, ] == 1))        met.flow <- c(met.flow, rep(q, times = num.req))      }    }  }  possible.flows <- cbind(donors, recipients, met.flow)  #Remove all flows involving the two invaders  possible.flows <- possible.flows[possible.flows[,2] != num.all.taxa - 1, ]  possible.flows <- possible.flows[possible.flows[,1] != num.all.taxa - 1, ]  possible.flows <- possible.flows[possible.flows[,2] != num.all.taxa, ]  possible.flows <- possible.flows[possible.flows[,1] != num.all.taxa, ]    real.flows <- possible.flows[sample(seq(1, dim(possible.flows)[1]), ceiling(direct.prop*dim(possible.flows)[1])), ]  real.flows <- as.matrix(real.flows)  dim(real.flows)    #Put flows into an array of num.taxa x num.taxa x num.mets   flow.array <- array(0, dim = c(num.all.taxa, num.all.taxa, num.mets.all))  donor.array <- array(0, dim = c(num.all.taxa, num.all.taxa, num.mets.all))  for(which.met in 1:num.mets.all){    donor.array[real.flows[, 1][real.flows[, 3] == which.met], real.flows[, 2][real.flows[, 3] == which.met], which.met] <- -1    flow.row <- real.flows[, 2][real.flows[, 3] == which.met]    flow.col <- real.flows[, 1][real.flows[, 3] == which.met]    for(pair in 1:length(flow.row)){      flow.array[flow.row[pair], flow.col[pair], which.met] <- 1    }  }    ## Variables   abun.series <- vector()  mets.series <- vector()    ##  Initialize vectors  init.abun <- 50  abuns <- c(rep(init.abun, num.taxa ), 0, 0)  mets.pool <- rep(0, num.mets.all)  met.stores <- matrix(0, num.mets.all, num.all.taxa)    # pull competitors from a normal distribution, but make sure draws are not too close together  # if competition values are extremely close together, model can take a very long time to equilibrate  min.space <- .003  min.comp.dist <- .001  comp.orig <- c(rnorm(num.taxa, comp.mean, comp.sd), inv.comp)  comp.nat <- comp.orig    while(min.comp.dist < min.space){    comp.dist <- as.matrix(dist(comp.nat))    diag(comp.dist) <- 1    comp.dist[upper.tri(comp.dist)] <- 1    which.below <- unique(which(comp.dist < min.space, arr.ind = T)[, 2])    comp.nat[which.below] <- rnorm(length(which.below), comp.mean, comp.sd)    min.comp.dist <- min(comp.dist)  }  #par(mfrow = c(2, 1))  #hist(comp.orig, xlim = range(c(comp.nat, comp.orig)), breaks = 10)  #hist(comp.nat, xlim = range(c(comp.nat, comp.orig)), breaks = 10)    comp.space <- comp.nat[-length(comp.nat)]    comp.vec <- c(comp.space, inv.comp, inv.comp)  #rnorm(num.all.taxa, comp.mean, comp.sd)  comp.vec <- apply(cbind(comp.vec, rep(min.comp, times = length(comp.vec))), 1, max)  if(stoch.comp) {    comp <- matrix(rep(comp.vec, num.mets.all), ncol = num.all.taxa, byrow = T)  } else {    comp <- matrix(rep(c(rep(comp.mean, times = num.taxa), inv.comp, inv.comp), num.mets.all), ncol = num.all.taxa, byrow = T)  }    dim(comp)    stop.val <- 0.001  max.abun.change <- 1  i <- 1  inv.added <- F  first.inv.at.second <- F  second.inv.added <- F  second.inv.control <- F  over.time <- F  over.time.half <- F  other.fail <- F    while(!(max.abun.change <= stop.val | over.time | other.fail)){ #if either of these are true, loop stops     mets.pool <- mets.pool  +  (input.rate * mets.in)        demand.mat <-  (reqs * comp)  *  ( matrix(rep(abuns, each = num.mets.all), num.mets.all, num.all.taxa) - met.stores )     demand <-  rowSums ( (reqs * comp)  *  ( matrix(rep(abuns, each = num.mets.all), num.mets.all, num.all.taxa) - met.stores ) )        comp.abil <- (matrix(rep(abuns, each = num.mets.all), num.mets.all, num.all.taxa) - met.stores ) * reqs * comp        comp.abil.rel <- comp.abil / rowSums(comp.abil)    comp.abil.rel[is.nan(comp.abil.rel)] <- 0 #for cases where row sums are 0, will return infinity. should be 0        mets.add <- (apply(cbind(demand, mets.pool), 1, min)) * comp.abil.rel        met.stores <- met.stores  +  mets.add    mets.pool <- mets.pool - rowSums(mets.add)    mets.pool <- apply(cbind(rep(0, length(mets.pool)), mets.pool), 1, max)        #Find the amount of only metabolites that are required by each taxon    #req.met.stores <- matrix(met.stores[as.logical(reqs)] , max(mets.req), num.taxa)        #growth <- apply(req.met.stores, 2, min)    #growth[is.nan(growth)] <- 0        #Make loop to calculate min of requried mets    growth <- vector()    for(nt in 1:num.all.taxa){      growth.n <- min.of.n(met.stores[, nt], min.n = n.mets.for.taxa[nt])      growth <- c(growth, growth.n)    }        abuns <- abuns + growth         met.ex <- t( growth * t(ex))        met.stores <- (met.stores - t( growth * t(reqs)) )        lim.nut <- vector()    current.taxa <- which(abuns > 1)    position <- 1    for(tx in current.taxa){      lim.nut[position] <- paste(tx, which(reqs[, tx] == 1 & met.stores[, tx] == 0), sep = "." )      position <- position + 1    }        mets.traded <- vector()    for(met.i in 1:num.mets.all){      flow.norm <- sweep(flow.array[,, met.i], 2, colSums(flow.array[,, met.i]), '/')      flow.norm[is.na(flow.norm)] <- 0      flow.norm <- flow.norm * flow.strength      met.avail <- flow.norm %*% met.ex[met.i, ]      met.missing <- abuns - met.stores[met.i, ]      mets.transfer <- apply(cbind(met.avail, met.missing), 1, min)      met.stores[met.i, ] <- met.stores[met.i, ]  +  mets.transfer      mets.traded[met.i] <- sum(mets.transfer)    }        # Subtract exchanged mets from mets to be excreted    met.stores <- met.stores * (1 - flush)    mets.pool <- (mets.pool  +  rowSums(met.ex) - mets.traded) * (1 - flush)    mets.pool <- apply(cbind(rep(0, length(mets.pool)), mets.pool), 1, max)        #abuns <- apply(cbind(floor(abuns * (1 - flush)), rep(0, times = length(abuns))), 1, max) #makes taxa go extinct     abuns <- abuns * (1 - flush)        abun.series <- cbind(abun.series, abuns)    mets.series <- cbind(mets.series, mets.pool)        #if(i %% 500 == 0) abuns[abuns > 1] <- sample(abuns[abuns > 1]) #perturb the system to see if it is stable        if(i > 1) max.abun.change <- max(abs(abuns - abun.series[, i - 1]))        # after initial equilibration, add an invader    eq.scenario <- inv.added == F & max.abun.change < stop.val        half.time <- (i == time.steps / 2) & inv.added == F    if(eq.scenario | half.time){      num.coex.half <- sum(abuns > 1)      #if(num.coex.half == 0) stop()      eq.mets.half <-  mets.pool       mets.all.preinv[run] <-  sum(mets.pool)      final.abuns.half <- abuns      met.stores.half <- met.stores      abuns[length(abuns) - 1] <- init.abun      mets.pool.prop <- mets.pool / sum(mets.pool)      mets.pool.prop <- mets.pool.prop[mets.pool.prop > 0]      mets.div[run] <- -1 * sum( mets.pool.prop * (log(mets.pool.prop)) )      mets.ev[run] <- mets.div[run] / log(num.coex.half)      avg.comp.pre[run] <- mean(comp.vec[1:num.taxa][abuns[1:num.taxa] > 1])      inv.added <- T      inv.add.time[run] <- i      max.abun.change <- init.abun #makes it so loop continues      ## record number of flows per metabolite per taxon      pres.taxa  <- which(abuns > 1)      flows.per[run] <- sum(real.flows[, 1] %in% pres.taxa & real.flows[, 2] %in% pres.taxa ) / (num.coex.half * mets.req)      flows.per.extra[run] <- sum(real.flows[, 1] %in% pres.taxa & real.flows[, 2] %in% pres.taxa ) / ((num.coex.half - mets.req) * mets.req)      num.mets.trade[run] <- sum(mets.traded)            realized.flows <- real.flows[real.flows[, 1] %in% pres.taxa & real.flows[, 2] %in% pres.taxa, ]      realized.flows <- matrix(realized.flows, ncol = 3)      if(dim(realized.flows)[1] > 0 ) {        recip.met.combo <- paste(realized.flows[, 2], realized.flows[, 3], sep = ".")        recip.met.agg <- tapply(recip.met.combo, recip.met.combo, length)        redundancy[run] <- mean(recip.met.agg)        redun.lim[run] <- mean(as.numeric(lapply(lim.nut, matches.in.vec, vec = recip.met.combo )))        flows.per.taxon[run] <- mean(tapply(realized.flows[, 2], realized.flows[, 2], length)[tapply(realized.flows[, 2], realized.flows[, 2], length) > 0])      } else {        redundancy[run] <- 0        flows.per.taxon[run] <- 0        redun.lim[run] <- 0      }            #Find redundancy in only limiting nutrient trades                  if(i == time.steps / 2) {        over.step.half <- over.step.half + 1      }    }        # if invader is added and model equilibrates or first invader goes extinct, add second invader     eq.scenario.v1 <- inv.added == T & max.abun.change < stop.val & second.inv.added == F        if(eq.scenario.v1 ){       #Update whether first invader succeeded      if(abuns[length(abuns) - 1] > 1) {        inv.persist[run] <-  1      } else {        inv.persist[run] <-  0      }            ## Record model values after first equilibrium reached       # How to change the model to discard runs where invader fails and to discard runs where it doesn't equilibrate soon enough      final.abuns[, run] <-  abuns      num.coex[run] <- sum(abuns > 1)      eq.mets[, run] <-  mets.pool      mets.all[run] <-  sum(mets.pool)      avg.comp.post.inv[run] <- mean(comp.vec[1:num.taxa][abuns[1:num.taxa] > 1])            #Record flows per metabolite after invader      pres.taxa.post  <- which(abuns > 1)      flows.per.post[run] <-  sum(real.flows[, 1] %in% pres.taxa.post & real.flows[, 2] %in% pres.taxa.post ) / (num.coex[run] * mets.req)      if(num.coex[run] == 0) flows.per.post[run] <- 0      realized.flows.post <- real.flows[real.flows[, 1] %in% pres.taxa.post & real.flows[, 2] %in% pres.taxa.post, ]      realized.flows.post <- matrix(realized.flows.post, ncol = 3)      if(dim(realized.flows.post)[1] > 0 ) {        recip.met.combo.post <- paste(realized.flows.post[, 2], realized.flows.post[, 3], sep = ".")        redundancy.post[run] <- mean(tapply(recip.met.combo.post, recip.met.combo.post, length))        redun.lim.post[run] <- mean(as.numeric(lapply(lim.nut, matches.in.vec, vec = recip.met.combo.post )))        flows.per.taxon.post[run] <- mean(tapply(realized.flows.post[, 2], realized.flows.post[, 2], length)[tapply(realized.flows.post[, 2], realized.flows.post[, 2], length) > 0])      } else {        redundancy.post[run] <- 0        flows.per.taxon.post[run] <- 0        redun.lim.post[run] <- 0      }            mets.pool.post <- mets.pool / sum(mets.pool)      mets.pool.post <- mets.pool.post[mets.pool.post > 0]      mets.div.post[run] <- -1 * sum(mets.pool.post * log(mets.pool.post))            ## Record changes in community structure after invader added      div.change[run] <- num.coex[run] - num.coex.half      inds.change[run] <- sum(abuns) - sum(final.abuns.half)      mets.change[run] <- sum(eq.mets.half) - sum(mets.pool)      mets.trade.change[run] <- sum(mets.traded) - sum(num.mets.trade[run])      flows.per.change[run] <- flows.per.post[run] - flows.per[run]            #record abundance of invader      inv.eq.abun[run] <- abuns[length(abuns) - 1]      # Add in second invader       abuns[length(abuns) ] <- init.abun      # Change max.abun.change so that loop continues      max.abun.change <- 1      # Record second inv added       second.inv.added <- T      #Record time when second inv added       time.second.inv <- i      #record time when second inv added      second.inv.add.time[run] <- i      #Record whether first inv was persisting at time of adding second inv       first.inv.at.second <- abuns[length(abuns) - 1] > 1          }            # See if second invader would have succeeded without first invader    if(inv.added == T & second.inv.added == T & second.inv.control== F & max.abun.change < stop.val){      #Update whether sequential second invader succeeded      if(abuns[length(abuns)] > 1) {        second.inv.persist[run] <-  1      } else {        second.inv.persist[run] <-  0      }      #Reset conditions back to when the first invader was added      met.stores <- met.stores.half      mets.pool <- eq.mets.half      abuns <- final.abuns.half      abuns[length(abuns)] <- init.abun      max.abun.change <- init.abun      second.inv.control <- T    }        if(i >= time.steps) {      over.time <- T      over.step.full <- over.step.full + 1    }        # if(i == 1) {    #   plot(abuns ~ rep(i, times = length(abuns)), xlim = c(0, 5000), pch = 20, col = seq(1, length(abuns), 1), ylim = c(0, 300))    #     } else {    #     points(abuns ~ rep(i, times = length(abuns)), pch = 20, col = seq(1, length(abuns), 1) )    #    }        i <- i  +  1     }    # seq.plot <- round(seq(1, dim(abun.series)[2], length.out = 400))  # par(mfrow = c(1, 1))  # matplot(t(abun.series[, seq.plot]), type = "l", main = paste(get("run"), get("i"), sep = "_") )    num.coex.preinv[run] <- num.coex.half  eq.mets.preinv[, run] <- eq.mets.half   final.abuns.preinv[, run] <-  final.abuns.half    abun.pa <- abun.series[, i - 1]  abun.pa[abun.pa >= 1] <- 1  abun.pa[abun.pa < 1] <- 0  second.inv.control.persist[run] <- abun.pa[length(abun.pa)]  inv.control.eq.abun[run] <- abuns[length(abuns) ]      if(over.time | over.time.half ) {    run <- run     over.step.count <- over.step.count + 1  } else {    run <- run + 1  }    # if(run %% 10 == 0 ) print(c(run, i) )  # print(c(run, i, st) )  }params <- c(input.rate, num.taxa, num.mets.all, mets.req, inv.mets.req, mets.excreted, flush, time.steps, num.runs, comp.mean, comp.sd, flow.strength)names(params) <- c("input.rate", "num.taxa", "num.mets.all", "mets.req", "inv.mets.req", 'mets.ex', "flush", "time.steps", "num.runs", "comp.mean", "comp.sd", "flow.strength")paramsover.step.prop <- over.step.count / (num.runs + over.step.count)#Create table of results from all the runssummary.results <- cbind(rep(comp.mean, times = num.runs), rep(direct.prop, times = num.runs), num.coex.preinv, num.coex, colSums(final.abuns.preinv), colSums(final.abuns), colSums(eq.mets.preinv), colSums(eq.mets), inv.persist, second.inv.persist, second.inv.control.persist, mets.div, mets.div.post)colnames(summary.results) <- c("comp.mean", "direct.prop", "num.coex.preinv", "num.coex.post", "total.ind.preinv", "total.ind.post", "total.mets.pre", "total.mets.post", "inv.persist", "second.inv.persist", "second.inv.control.persist", "metabolite.diversity", "metabolite.diversity.post")rownames(summary.results) <- seq(1, dim(summary.results)[1], 1)#Create vector to hold summary statistics (means, medians, IQRs) of variables of interestsummary.stats <- c(apply(summary.results[, 1:8], 2, median), mean(summary.results[, 9]), mean(summary.results[, 10]), mean(summary.results[, 11]), median(summary.results[, 12]), median(summary.results[,13]),  over.step.prop)#summary.stats <- c(apply(summary.results[, 1:8], 2, mean), mean(summary.results[, 9]), mean(summary.results[, 10]), over.step.prop)summary.stats <- c(summary.stats, quantile(summary.results[,3], probs = c(0.25, 0.75)), quantile(summary.results[,5], probs = c(.25, .75)), quantile(summary.results[,7], probs = c(.25, .75)), quantile(summary.results[, 12], probs = c(.25, .75)))summary.stats <- c(summary.stats, median(summary.results[,5]/summary.results[,7]), quantile(summary.results[,5] / summary.results[,7], probs = c(.25, .75))) names(summary.stats) <- c(colnames(summary.results), "over.step.prop", "IQRlowCoex", "IQRhighCoex", "IQRlowInd", "IQRhighInd", "IQRlowMets", "IQRhighMets", "IQRlowMetDiv", "IQRhighMetDiv",  "Effic", "IQRlowEffic", "IQRhighEffic")#Add median and IQR of num.coex and inds and mets for communities where invaders failed versus succeedednum.coex.succeed <- summary.results[, 3][summary.results[, 9] == 1]num.coex.fail <- summary.results[, 3][summary.results[, 9] == 0]num.coex.post.succeed <- summary.results[, 4][summary.results[,9] == 1]inds.succeed <- summary.results[, 5][summary.results[, 9] == 1]inds.fail <- summary.results[, 5][summary.results[, 9] == 0]inds.post.succeed <- summary.results[,6][summary.results[,9] == 1]mets.succeed <- summary.results[, 7][summary.results[, 9] == 1]mets.fail <- summary.results[, 7][summary.results[, 9] == 0]mets.post.succeed <- summary.results[,8][summary.results[,9] == 0]met.div.succeed <- summary.results[,12][summary.results[,9] == 1]met.div.fail <- summary.results[,12][summary.results[,9] == 0]met.div.post.succeed <- summary.results[,13][summary.results[,9] ==0 ]fail.success.stats <- c(quantile(num.coex.fail, probs = c(.25, .5, .75)),                         quantile(num.coex.succeed, probs = c(.25, .5, .75)),                         quantile(inds.fail, probs = c(.25, .5, .75)),                         quantile(inds.succeed, probs = c(.25, .5, .75)) ,                         quantile(mets.fail, probs = c(.25, .5, .75)),                         quantile(mets.succeed, probs = c(.25, .5, .75)),                         quantile(met.div.succeed, probs = c(.25, .5, .75)),                         quantile(met.div.fail, probs = c(.25, .5, .75)) )names(fail.success.stats) <- c("num.coex.fail.25", "num.coex.fail.med", "num.coex.fail.75",                                "num.coex.succeed.25", "num.coex.succeed.med", "num.coex.succeed.75",                                "inds.fail.25", "inds.fail.med", "inds.fail.75",                                "inds.succeed.25", "inds.succeed.med", "inds.succeed.75",                                "mets.fail.25", "mets.fail.med", "mets.fail.75",                                "mets.succeed.25", "mets.succeed.med", "mets.succeed.75",                                "met.div.succeed.25", "met.div.succeed.med", "met.div.succeed.75",                                "met.div.fail.25", "met.div.fail.med", "met.div.fail.75")conditional.inv <- c(tapply(second.inv.persist, inv.persist, mean),                      tapply(second.inv.control.persist, inv.persist, mean))names(conditional.inv) <- c("second.inv.cond.fail", "second.ind.cond.succeed",                             "control.inv.cond.fail", "control.ind.cond.succeed")post.inv.stats <- c(quantile(num.coex.post.succeed, probs = c(.25, .5, .75)),                     quantile(inds.post.succeed, probs = c(.25, .5, .75)),                     quantile(mets.post.succeed, probs = c(.25, .5, .75)),                     quantile(met.div.post.succeed, probs = c(.25, .5, .75)))names(post.inv.stats) <- c("num.coex.post.succeed.25", "num.coex.post.succeed.med", "num.coex.post.succeed.75",                            "inds.post.succeed.25", "inds.post.succeed.med", "inds.post.succeed.75",                            "mets.post.succeed.25", "mets.post.succeed.med", "mets.post.succeed.75",                            "met.div.post.succeed.25", "met.div.post.succeed.med", "met.div.post.succeed.75")## Add information about flows per taxon flows.per.vec <- quantile(flows.per, probs = c(.25, .5, .75), na.rm = T)names(flows.per.vec) <- c("flows.per.25", "flows.per.med", "flows.per.75")## Add information about number of metabolites tradedmets.traded.vec <- quantile(num.mets.trade, probs = c(.25, .5, .75))names(mets.traded.vec) <- c("mets.traded.25", "mets.traded.med", "mets.traded.75")## Add information about the evennes sof metabolitesmets.ev.vec <- quantile(mets.ev, probs = c(.25, .5, .75) )names(mets.ev.vec) <- c("mets.ev.25", "mets.ev.med", "mets.ev.75")## Make vectors of connectivity metrics for uninvaded, invasible, and post invasionmets.traded.uninvasible <- num.mets.trade[inv.persist == 0]mets.traded.invasible <- num.mets.trade[inv.persist == 1]mets.traded.post.inv <- (num.mets.trade + mets.trade.change)[inv.persist == 1]mets.traded.by.invasibility <- c(quantile(mets.traded.uninvasible, probs = c(.25, .5, .75), na.rm = T) ,                                  quantile(mets.traded.invasible, probs = c(.25, .5, .75), na.rm = T) ,                                  quantile(mets.traded.post.inv, probs = c(.25, .5, .75), na.rm = T) )names(mets.traded.by.invasibility) <- c("mets.traded.uninvasible.25", "mets.traded.uninvasible.med", "mets.traded.uninvasible.75",                                         "mets.traded.invasible.25", "mets.traded.invasible.med", "mets.traded.invasible.75",                                        "mets.traded.post.inv.25", "mets.traded.post.inv.med", "mets.traded.post.inv.75")## Now for community structure parallels num.coex.uninvasible <- num.coex.preinv[inv.persist == 0]num.coex.invasible <- num.coex.preinv[inv.persist == 1]num.coex.post.inv <- num.coex[inv.persist == 1]num.coex.by.invasibility <- c( quantile(num.coex.uninvasible, probs = c(.25, .5, .75), na.rm = T) ,                                quantile(num.coex.invasible, probs = c(.25, .5, .75), na.rm = T) ,                                quantile(num.coex.post.inv, probs = c(.25, .5, .75), na.rm = T) )names(num.coex.by.invasibility) <- c("num.coex.uninvasible.25", "num.coex.uninvasible.med", "num.coex.uninvasible.75",                                      "num.coex.invasible.25", "num.coex.invasible.med", "num.coex.invasible.75",                                     "num.coex.post.inv.25", "num.coex.post.inv.med", "num.coex.post.inv.75")total.inds.uninvasible <- colSums(final.abuns.preinv)[inv.persist == 0]total.inds.invasible <- colSums(final.abuns.preinv)[inv.persist == 1]total.inds.post.inv <- colSums(final.abuns)[inv.persist == 1]total.inds.by.invasibility <- c( quantile(total.inds.uninvasible, probs = c(.25, .5, .75), na.rm = T) ,                                  quantile(total.inds.invasible, probs = c(.25, .5, .75), na.rm = T) ,                                  quantile(total.inds.post.inv, probs = c(.25, .5, .75), na.rm = T) )names(total.inds.by.invasibility) <- c("total.inds.uninvasible.25", "total.inds.uninvasible.med", "total.inds.uninvasible.75",                                        "total.inds.invasible.25", "total.inds.invasible.med", "total.inds.invasible.75",                                       "total.inds.post.inv.25", "total.inds.post.inv.med", "total.inds.post.inv.75")met.pools.uninvasible <- colSums(eq.mets.preinv)[inv.persist == 0]met.pools.invasible <- colSums(eq.mets.preinv)[inv.persist == 1]met.pools.post.inv <- colSums(eq.mets)[inv.persist == 1]met.pools.by.invasibility <- c( quantile(met.pools.uninvasible, probs = c(.25, .5, .75), na.rm = T) ,                                 quantile(met.pools.invasible, probs = c(.25, .5, .75), na.rm = T) ,                                 quantile(met.pools.post.inv, probs = c(.25, .5, .75), na.rm = T) )names(met.pools.by.invasibility) <- c("met.pools.uninvasible.25", "met.pools.uninvasible.med", "met.pools.uninvasible.75",                                       "met.pools.invasible.25", "met.pools.invasible.med", "met.pools.invasible.75",                                      "met.pools.post.inv.25", "met.pools.post.inv.med", "met.pools.post.inv.75")comp.avg.uninvasible <- avg.comp.pre[inv.persist == 0]comp.avg.invasible <- avg.comp.pre[inv.persist == 1]comp.avg.post.inv <- avg.comp.post.inv[inv.persist == 1]comp.avg.by.invasibility <- c( quantile(comp.avg.uninvasible, probs = c(.25, .5, .75), na.rm = T) ,                                quantile(comp.avg.invasible, probs = c(.25, .5, .75), na.rm = T) ,                                quantile(comp.avg.post.inv, probs = c(.25, .5, .75), na.rm = T) )names(comp.avg.by.invasibility) <- c("comp.avg.uninvasible.25", "comp.avg.uninvasible.med", "comp.avg.uninvasible.75",                                      "comp.avg.invasible.25", "comp.avg.invasible.med", "comp.avg.invasible.75",                                     "comp.avg.post.inv.25", "comp.avg.post.inv.med", "comp.avg.post.inv.75")## Look at how average competition coefficients change after invasionmean(avg.comp.pre)mean(avg.comp.post.inv)avg.comp.change <- avg.comp.post.inv - avg.comp.pre## Add in redundancy.lim metrics redundancy.lim.invasible <- redun.lim[inv.persist == 1]redundancy.lim.post.invaded <- redun.lim.post[inv.persist == 1]redundancy.lim.uninvasible <- redun.lim[inv.persist == 0]redundancy.lim.change.invaded <- redun.lim.post[inv.persist == 1] - redun.lim[inv.persist == 1]redundancy.lim.second.inv.succeed <- redun.lim[inv.persist == 1 & second.inv.persist == 1]redundancy.lim.second.inv.fail <- redun.lim[inv.persist == 1 & second.inv.persist == 0]redundancy.lim.post.second.inv.succeed <- redun.lim.post[inv.persist == 1 & second.inv.persist == 1]redundancy.lim.post.second.inv.fail <- redun.lim.post[inv.persist == 1 & second.inv.persist == 0]redundancy.lim.vec <- c(quantile(redundancy.lim.invasible, probs = c(.25, .5, .75), na.rm =  T),                         quantile(redundancy.lim.post.invaded, probs = c(.25, .5, .75), na.rm =  T),                         quantile(redundancy.lim.uninvasible, probs = c(.25, .5, .75), na.rm =  T),                         quantile(redundancy.lim.change.invaded, probs = c(.25, .5, .75), na.rm =  T),                         quantile(redundancy.lim.second.inv.succeed, probs = c(.25, .5, .75), na.rm =  T),                        quantile(redundancy.lim.second.inv.fail, probs = c(.25, .5, .75), na.rm =  T),                        quantile(redundancy.lim.post.second.inv.succeed, probs = c(.25, .5, .75), na.rm =  T),                        quantile(redundancy.lim.post.second.inv.fail, probs = c(.25, .5, .75), na.rm =  T))names(redundancy.lim.vec) <- c("redundancy.lim.invasible.25", "redundancy.lim.invasible.med", "redundancy.lim.invasible.75",                                "redundancy.lim.post.invaded.25", "redundancy.lim.post.invaded.med", "redundancy.lim.post.invaded.75",                               "redundancy.lim.uninvasible.25", "redundancy.lim.uninvasible.med", "redundancy.lim.uninvasible.75",                               "redundancy.lim.change.invaded.25", "redundancy.lim.change.invaded.med", "redundancy.lim.change.invaded.75",                                "redundancy.lim.second.inv.succeed.25", "redundancy.lim.second.inv.succeed.med", "redundancy.lim.second.inv.succeed.75",                                "redundancy.lim.second.inv.fail.25", "redundancy.lim.second.inv.fail.med", "redundancy.lim.second.inv.fail.75",                                "redundancy.lim.post.second.inv.succeed.25", "redundancy.lim.post.second.inv.succeed.med", "redundancy.lim.post.second.inv.succeed.75",                                "redundancy.lim.post.second.inv.fail.25", "redundancy.lim.post.second.inv.fail.med", "redundancy.lim.post.second.inv.fail.75")## Add median and IQR for how community structure changes in response to invasion## First, pick out only communities where invader was successfulinvaded.num.coex.change <- div.change[inv.persist == 1]invaded.mets.change <- mets.change[inv.persist == 1]invaded.inds.change <- inds.change[inv.persist == 1]invaded.traded.change <- mets.trade.change[inv.persist == 1]invaded.flows.change <- flows.per.change[inv.persist == 1]invaded.mets.div.change <- (mets.div.post - mets.div)[inv.persist == 1]inv.change.stats <- c(quantile(invaded.num.coex.change, probs = c(.25, .5, .75), na.rm = T),                       quantile(invaded.mets.change, probs = c(.25, .5, .75), na.rm = T),                       quantile(invaded.inds.change, probs = c(.25, .5, .75), na.rm = T),                       quantile(invaded.traded.change, probs = c(.25, .5, .75), na.rm = T),                       quantile(invaded.flows.change, probs = c(.25, .5, .75), na.rm = T),                       quantile(invaded.mets.div.change, probs = c(.25, .5, .75), na.rm = T) )names(inv.change.stats) <- c("invaded.num.coex.change.25", "invaded.num.coex.change.med", "invaded.num.coex.change.75",                              "invaded.mets.change.25", "invaded.mets.change.med", "invaded.mets.change.75",                              "invaded.inds.change.25", "invaded.inds.change.med", "invaded.inds.change.75",                              "invaded.traded.change.25", "invaded.traded.change.med", "invaded.traded.change.75",                              "invaded.flows.change.25", "invaded.flows.change.med", "invaded.flows.change.75",                              "invaded.mets.div.change.25", "invaded.mets.div.change.med", "invaded.mets.div.change.75")inv.change.means <- c(mean(invaded.num.coex.change, na.rm = T),                       mean(invaded.mets.change ,na.rm = T),                       mean(invaded.inds.change, na.rm = T),                       mean(invaded.traded.change, na.rm = T),                       mean(invaded.flows.change, na.rm = T),                       mean(invaded.mets.div.change ,na.rm = T),                       mean(redundancy.lim.change.invaded, na.rm = T))names(inv.change.means) <- c("invaded.num.coex.mean", "invaded.mets.change.mean", "invaded.inds.change.mean", "invaded traded.change.mean", "invaded.flows.change.mean", "invaded.mets.div.change.mean", "invaded.redundancy.lim.change.mean")## Record change in structure split by whether second invader was successful or notsecond.success.coex.change <-  div.change[inv.persist == 1 & second.inv.persist == 1]second.fail.coex.change <- div.change[inv.persist == 1 & second.inv.persist == 0]second.success.inds.change <-  inds.change[inv.persist == 1 & second.inv.persist == 1]second.fail.inds.change <- inds.change[inv.persist == 1 & second.inv.persist == 0]second.success.mets.change <-  mets.change[inv.persist == 1 & second.inv.persist == 1]second.fail.mets.change <- mets.change[inv.persist == 1 & second.inv.persist == 0]#Record change in connectivity split by whether second invader was successful or notsecond.success.traded.change <-  mets.trade.change[inv.persist == 1 & second.inv.persist == 1]second.fail.traded.change <- mets.trade.change[inv.persist == 1 & second.inv.persist == 0]second.success.flows.change <-  flows.per.change[inv.persist == 1 & second.inv.persist == 1]second.fail.flows.change <- flows.per.change[inv.persist == 1 & second.inv.persist == 0]second.inv.cond.stats <- c(quantile(second.success.coex.change, probs = c(.25, .5, .75), na.rm = T),                           quantile(second.fail.coex.change, probs = c(.25, .5, .75), na.rm = T),                            quantile(second.success.inds.change, probs = c(.25, .5, .75), na.rm = T),                            quantile(second.fail.inds.change, probs = c(.25, .5, .75), na.rm = T),                            quantile(second.success.mets.change, probs = c(.25, .5, .75), na.rm = T),                            quantile(second.fail.mets.change, probs = c(.25, .5, .75), na.rm = T),                            quantile(second.success.traded.change, probs = c(.25, .5, .75), na.rm = T),                            quantile(second.fail.traded.change, probs = c(.25, .5, .75), na.rm = T),                            quantile(second.success.flows.change, probs = c(.25, .5, .75), na.rm = T),                            quantile(second.fail.flows.change, probs = c(.25, .5, .75), na.rm = T)  )names(second.inv.cond.stats) <- c("second.success.coex.change.25", "second.success.coex.change.med", "second.success.coex.change.75",                                   "second.fail.coex.change.25", "second.fail.coex.change.med", "second.fail.coex.change.75",                                   "second.success.inds.change.25", "second.success.inds.change.med", "second.success.inds.change.75",                                   "second.fail.inds.change.25", "second.fail.inds.change.med", "second.fail.inds.change.75",                                   "second.success.mets.change.25", "second.success.mets.change.med", "second.success.mets.change.75",                                   "second.fail.mets.change.25", "second.fail.mets.change.med", "second.fail.mets.change.75",                                   "second.success.traded.change.25", "second.success.traded.change.med", "second.success.traded.change.75",                                   "second.fail.traded.change.25", "second.fail.traded.change.med", "second.fail.traded.change.75",                                   "second.success.flows.change.25", "second.success.flows.change.med", "second.success.flows.change.75",                                   "second.fail.flows.change.25", "second.fail.flows.change.med", "second.fail.flows.change.75")second.inv.cond.means <- c(mean(second.success.coex.change),                           mean(second.fail.coex.change),                            mean(second.success.inds.change),                            mean(second.fail.inds.change),                            mean(second.success.mets.change),                            mean(second.fail.mets.change),                            mean(second.success.traded.change),                            mean(second.fail.traded.change),                            mean(second.success.flows.change),                            mean(second.fail.flows.change) )second.inv.cond.sd <- c(sd(second.success.coex.change),                        sd(second.fail.coex.change),                         sd(second.success.inds.change),                         sd(second.fail.inds.change),                         sd(second.success.mets.change),                         sd(second.fail.mets.change),                         sd(second.success.traded.change),                         sd(second.fail.traded.change),                         sd(second.success.flows.change),                         sd(second.fail.flows.change) )## Add in redundancy metrics redundancy.invasible <- redundancy[inv.persist == 1]redundancy.post.invaded <- redundancy.post[inv.persist == 1]redundancy.uninvasible <- redundancy[inv.persist == 0]redundancy.change.invaded <- redundancy.post[inv.persist == 1] - redundancy[inv.persist == 1]redundancy.second.inv.succeed <- redundancy[inv.persist == 1 & second.inv.persist == 1]redundancy.second.inv.fail <- redundancy[inv.persist == 1 & second.inv.persist == 0]redundancy.post.second.inv.succeed <- redundancy.post[inv.persist == 1 & second.inv.persist == 1]redundancy.post.second.inv.fail <- redundancy.post[inv.persist == 1 & second.inv.persist == 0]redundancy.vec <- c(quantile(redundancy.invasible, probs = c(.25, .5, .75), na.rm =  T),                     quantile(redundancy.post.invaded, probs = c(.25, .5, .75), na.rm =  T),                     quantile(redundancy.uninvasible, probs = c(.25, .5, .75), na.rm =  T),                     quantile(redundancy.change.invaded, probs = c(.25, .5, .75), na.rm =  T),                     quantile(redundancy.second.inv.succeed, probs = c(.25, .5, .75), na.rm =  T),                    quantile(redundancy.second.inv.fail, probs = c(.25, .5, .75), na.rm =  T),                    quantile(redundancy.post.second.inv.succeed, probs = c(.25, .5, .75), na.rm =  T),                    quantile(redundancy.post.second.inv.fail, probs = c(.25, .5, .75), na.rm =  T))names(redundancy.vec) <- c("redundancy.invasible.25", "redundancy.invasible.med", "redundancy.invasible.75",                            "redundancy.post.invaded.25", "redundancy.post.invaded.med", "redundancy.post.invaded.75",                           "redundancy.uninvasible.25", "redundancy.uninvasible.med", "redundancy.uninvasible.75",                           "redundancy.change.invaded.25", "redundancy.change.invaded.med", "redundancy.change.invaded.75",                            "redundancy.second.inv.succeed.25", "redundancy.second.inv.succeed.med", "redundancy.second.inv.succeed.75",                            "redundancy.second.inv.fail.25", "redundancy.second.inv.fail.med", "redundancy.second.inv.fail.75",                            "redundancy.post.second.inv.succeed.25", "redundancy.post.second.inv.succeed.med", "redundancy.post.second.inv.succeed.75",                            "redundancy.post.second.inv.fail.25", "redundancy.post.second.inv.fail.med", "redundancy.post.second.inv.fail.75")redundancy.pre <- c(quantile(redun.lim, probs = c(.25, .5, .75), na.rm = T))names(redundancy.pre) <- c("redun.lim.25", "redun.lim.med" ,"redun.lim.75")## Create vector of means in addition to mediansmean.vec <- c(mean(num.coex.preinv, na.rm = T),               mean(summary.results[, 5], na.rm = T),               mean(eq.mets.preinv, na.rm = T),               mean(num.mets.trade, na.rm = T),               mean(flows.per.taxon, na.rm = T),               mean(redun.lim, na.rm = T))names(mean.vec) <- c("num.coex.mean", "total.inds.mean", "eq.mets.mean", "mets.traded.mean", "flows.per.tax.mean", "redun.lim.mean")## Split out results by uninvasible, invasible, and post invasionflows.per.uninvasible <- flows.per[inv.persist == 0]flows.per.invasible <- flows.per[inv.persist == 1]flows.per.post.inv <- flows.per.post[inv.persist == 1]second.success.flows.per.init <-  (flows.per)[inv.persist == 1 & second.inv.persist == 1]second.fail.flows.per.init <- (flows.per)[inv.persist == 1 & second.inv.persist == 0]second.success.flows.per.post <-  (flows.per.post)[inv.persist == 1 & second.inv.persist == 1]second.fail.flows.per.post <- (flows.per.post)[inv.persist == 1 & second.inv.persist == 0]flows.per.by.invasibility <- c( quantile(flows.per.uninvasible, probs = c(.25, .5, .75), na.rm = T) ,                                 quantile(flows.per.invasible, probs = c(.25, .5, .75), na.rm = T) ,                                 quantile(flows.per.post.inv, probs = c(.25, .5, .75), na.rm = T),                                quantile(second.success.flows.per.init, probs = c(.25, .5, .75), na.rm = T) ,                                quantile(second.fail.flows.per.init, probs = c(.25, .5, .75), na.rm = T),                                quantile(second.success.flows.per.post, probs = c(.25, .5, .75), na.rm = T) ,                                quantile(second.fail.flows.per.post, probs = c(.25, .5, .75), na.rm = T) )names(flows.per.by.invasibility) <- c("flows.per.uninvasible.25", "flows.per.uninvasible.med", "flows.per.uninvasible.75",                                       "flows.per.invasible.25", "flows.per.invasible.med", "flows.per.invasible.75",                                      "flows.per.post.inv.25", "flows.per.post.inv.med", "flows.per.post.inv.75",                                       "second.success.flows.per.init.25", "second.success.flows.per.init.med", "second.success.flows.per.init.75",                                       "second.fail.flows.per.init.25", "second.fail.flows.per.init.med", "second.fail.flows.per.init.75",                                       "second.success.flows.per.post.25", "second.success.flows.per.post.med", "second.success.flows.per.post.75",                                       "second.fail.flows.per.post.25", "second.fail.flows.per.post.med", "second.fail.flows.per.post.75")#### Combine all summary statistics into a single vector to write as a .csv summary.stats <- c(summary.stats, fail.success.stats, conditional.inv, post.inv.stats, flows.per.vec, mets.traded.vec, mets.ev.vec, num.coex.by.invasibility, total.inds.by.invasibility, met.pools.by.invasibility, mets.traded.by.invasibility, comp.avg.by.invasibility, redundancy.lim.vec, inv.change.means, inv.change.stats, second.inv.cond.stats, redundancy.vec, redundancy.pre, mean.vec, flows.per.by.invasibility)write.csv(t(summary.stats) , "MetModelOutput.csv")
