# Supplementary material for: Disruption of cross-feeding interactions by invading taxa can cause invasional meltdown in microbial communities
Source: Proc Biol Sci. 2020 May 13;287(1927):20192945. doi: 10.1098/rspb.2019.2945 (PMC7287355; doi:10.1098/rspb.2019.2945)
Supplement: Supplementary Online Materials [file rspb20192945supp2.pdf]

Supplementary Online Material: Sensitivity Analyses & Model Equations
These supplementary materials contain two sensitivity analyses to evaluate how strongly the central results in the main text depend on assumptions of the model. First, I investigated how the results change when the invader is able to cross-feed (Sensitivity Analysis # 1, pp. 1- 3). In this case, the invader is indistinguishable from native taxa, albeit with a relatively high competition value (though not higher than could be assigned by chance). Second, I allowed native taxa to have variable cross-feeding abilities, rather than assuming that metabolites were divided equally among all cross-feeders (Sensitivity Analysis # 2, pp. 4-6). In this case, I set cross-feeding ability as equal to competition values. For these two scenarios, I quantified the same outcomes as in the main text, and present them below. Additionally, the mathematical description of the model can be found in these supplementary materials, after the sensitivity analyses (pp. 7-9).

Sensitivity analysis 1: Invaders have the same cross-feeding abilities as native taxa

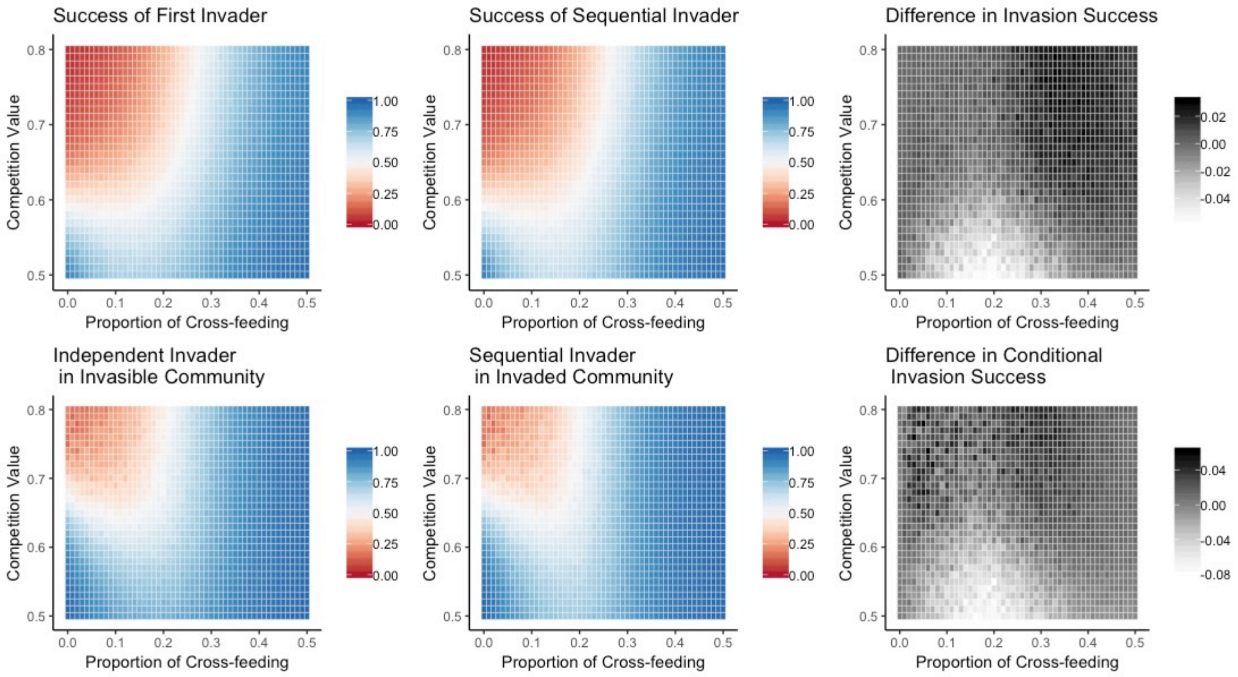

Fig. S1: Same results as presented in Fig. 2 in the main text, but under the condition that invaders have equivalent cross-feeding dynamics as native taxa.

Allowing invasive taxa to cross-feed substantially changes invasion success. When invaders can cross-feed, primary invasions are much more likely to be successful (upper left panel); in fact, higher rates of cross-feeding in the community facilitate the invader. Secondary invaders are, overall, slightly less successful than primary invaders (upper middle panel), and the greatest discrepancy between primary and secondary invaders occurs at intermediate levels of cross-feeding (upper right panel). This result that a primary invasion can make a later invasion more difficult is in direct contrast to the results presented in the main text. Thus, the cross-feeding dynamics of an introduced taxon has strong influence on both its ability to join the community and the potential for further taxa to join the community.

When looking only at communities that were successfully invaded, a different invader is generally also successful there (lower left panel). A secondary invader shows similar success patterns to a primary invader (lower middle panel), but there are regions of parameter space

where the secondary invader has both greater and lesser success (lower right panel). In general, when competition is low, secondary invaders were less successful, especially at intermediate levels of crossfeeding. However, when competition was high, a secondary invader was often more successful, due to the presence of the primary invader.

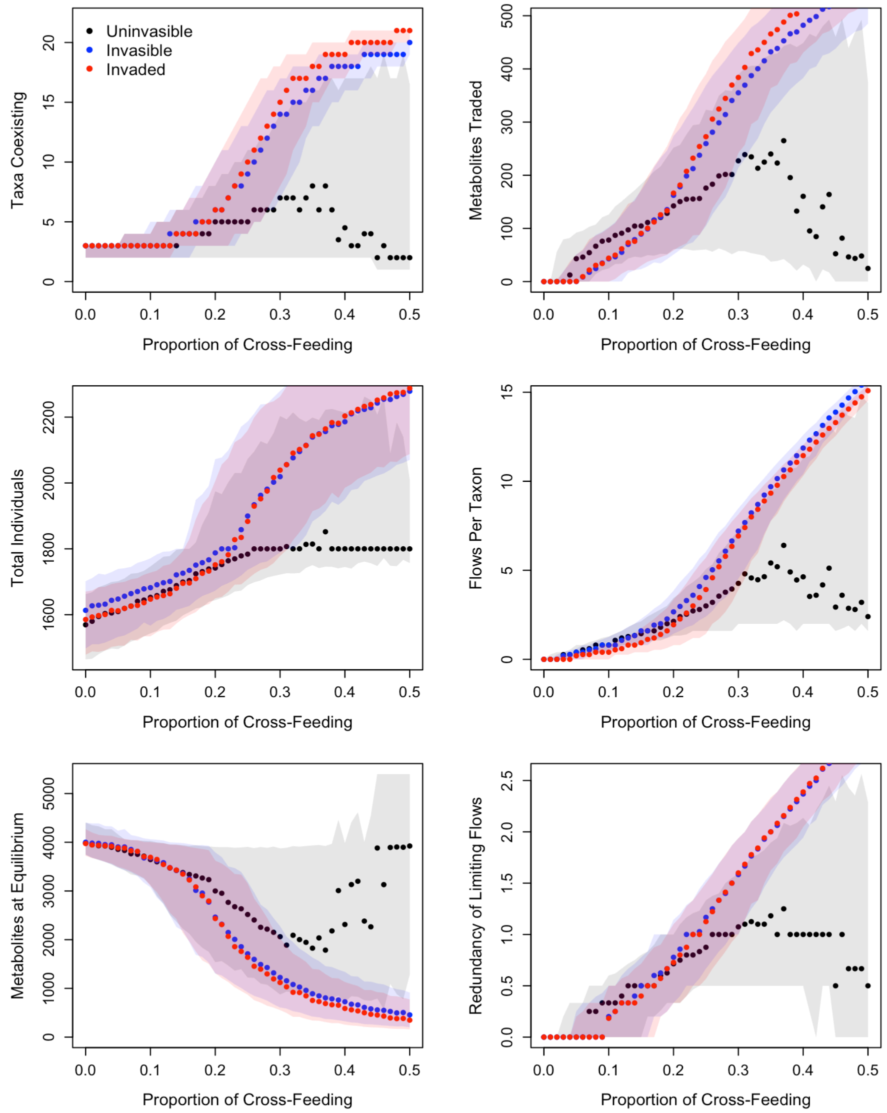

Fig. S2: Same results as presented in Fig. 4 in the main text. When invaders are able to cross-feed, a successful invasion only minimally changes community structure and metabolite exchange networks.

In contrast to Fig. 5 in the main text, which showed that the presence of an invader changes both community structure and metabolite exchange dynamics, Fig. S2 shows that the introduction of an invader generally has small effects on the communities. When native communities are already highly diverse (i.e. have a large number of taxa coexisting), the invader often simply joins the community, without dislodging other taxa (upper left panel). In these cases, the number of metabolites traded increases (upper right panel), as another taxon is added to the network of metabolite transfers.

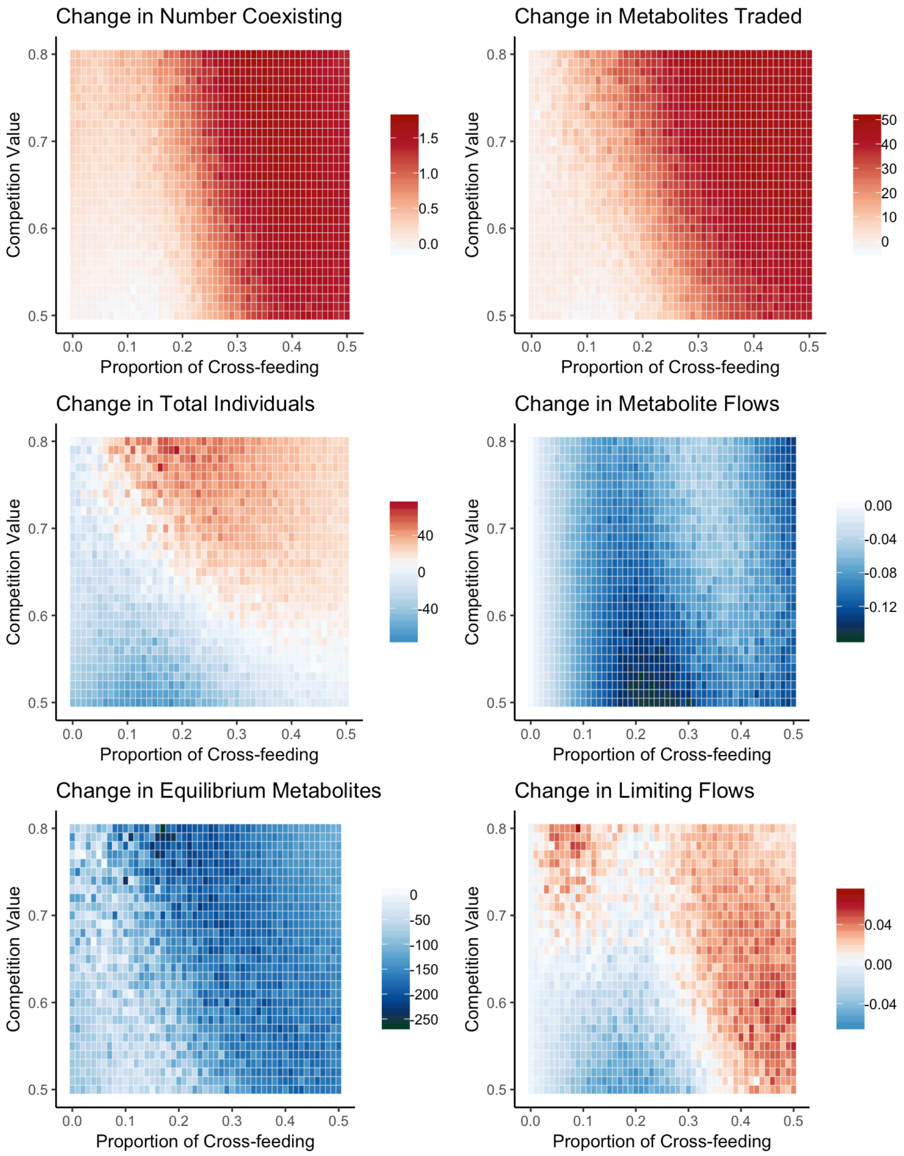

Fig. S3: Same results as presented in Fig. 5 in the main text. Warm colors indicate increases, and cool colors indicate decreases.

Allowing the invaders to cross-feed alters the impacts of a successful invasion on community structure and metabolite exchange. When invaders can cross-feed, they are much less likely to remove taxa from communities or diminish the number of metabolites exchanged within the community (upper panels). Additionally, there is less change in the total number of taxa in the community (left middle panel). Invading taxa also generally lead to decreases in the number of equilibrium metabolites (bottom left panel). Finally, invaders have a smaller impact on overall cross-feeding networks, although they do generally lead to slightly fewer cross-feeding relationships overall (right middle panel). However, the effect of invaders on the number of metabolite flows providing limiting nutrients could be either positive or negative, depending on the combination of cross-feeding and competition parameters (bottom right panel).

Sensitivity Analysis 2: Cross-feeding ability is equal to competitive ability

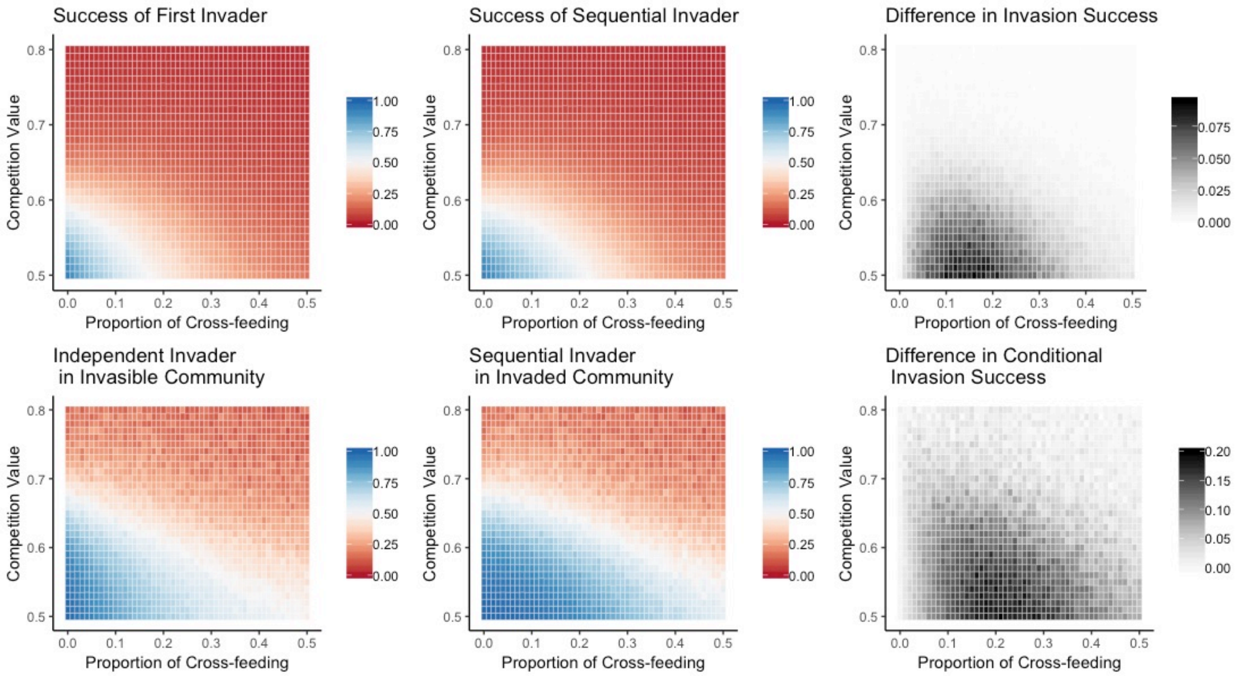

Fig. S4: Same results as presented in Fig. 2 in the main text, but under the condition that cross-feeding abilities are variable between taxa, being set as equal to each taxon's competition value.

In these simulations, the distinction from the model presented in the main text is that taxa in the native community have varying levels of cross-feeding abilities. However, this change has minimal impact on invasive taxa, as invasion success is largely unchanged.

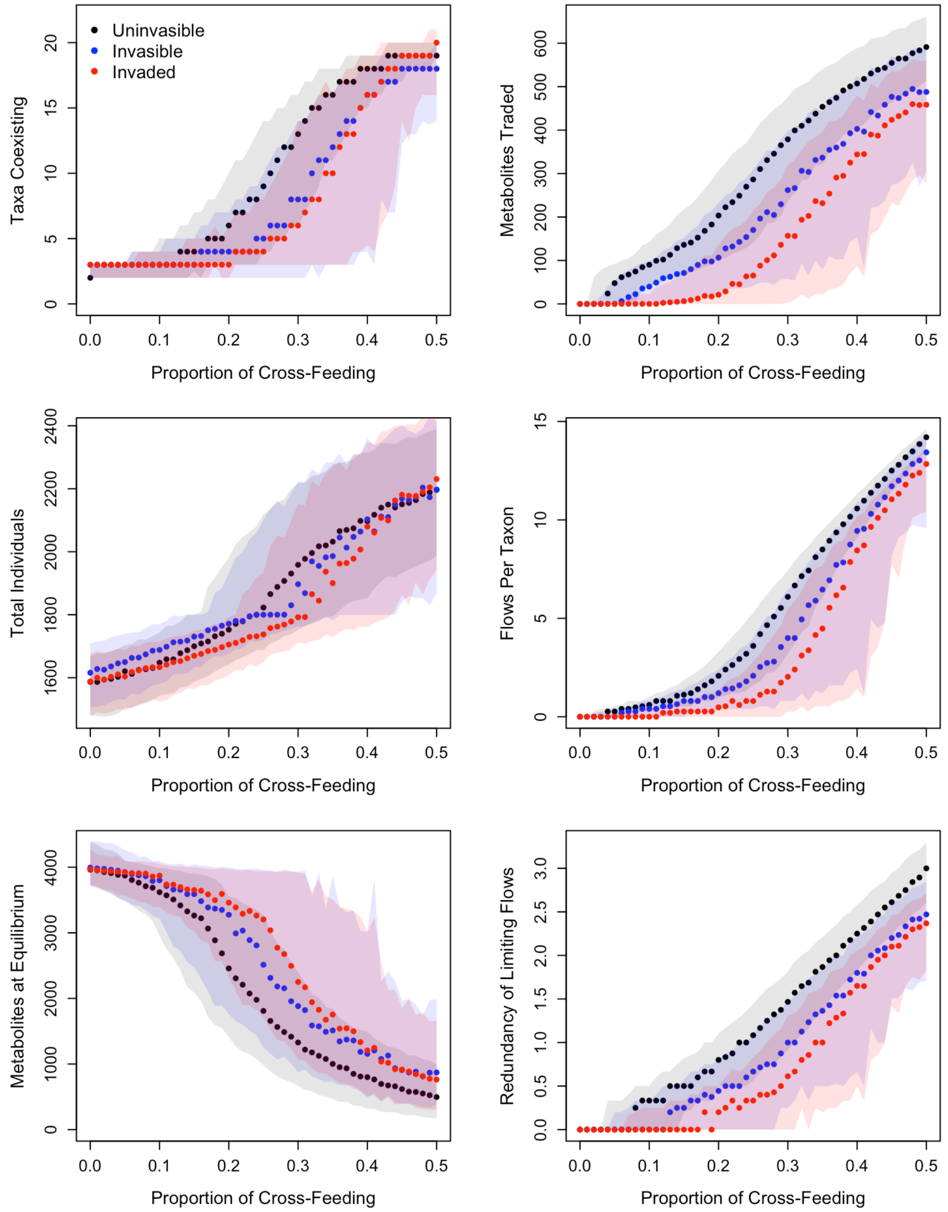

Fig. S5: Same results as presented in Fig. 4 in the main text. Allowing for variation of cross-feeding abilities in the native taxa has minimal effect on model results.

As with invasion success, changing the model to allow native taxa to be differentially good at cross-feeding shows minimal differences in community structure and metabolite transfer networks.

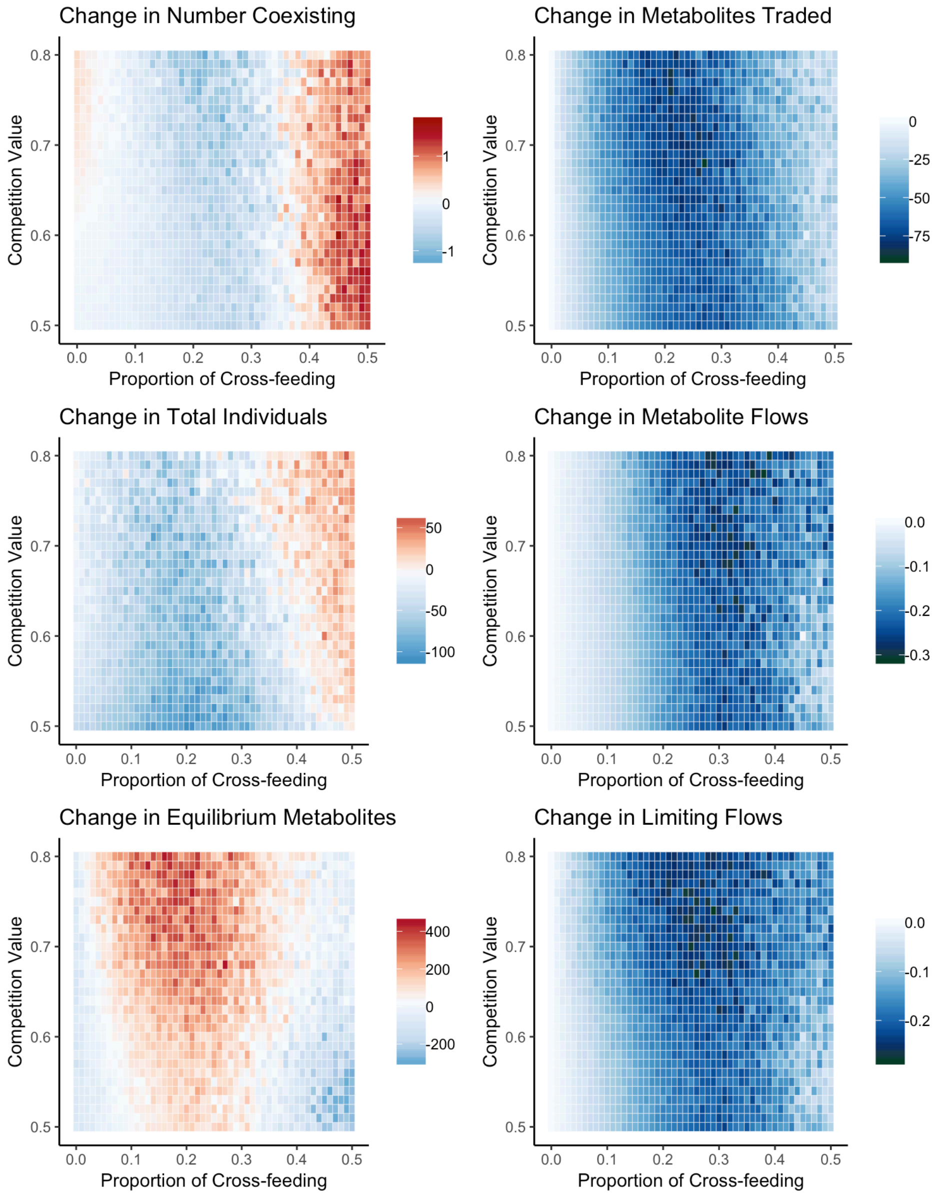

Fig. S6: Same results as presented in Fig. 6 in the main text.

Finally, Fig. S6 looks at how communities changed in response to a successful invader, when the native taxa varied in cross-feeding ability. These results are, again, largely similar to those presented in the main text, where there is no variation in cross-feeding ability.

These equations describe the dynamics of the native community before the invader is introduced. When the invader is added, the number of taxa increases by one, but cross-feeding is not affected, as the invader has no direct cross-feeding relationships. The model can be mathematically described as follows:

Vector and matrix definitions:

Y is a vector of the abundances of native taxa
$Y = (Y_1, Y_2, \ldots Y_x)$

Where $Y_j(t)$ denotes the abundance of taxon j at time t

c is a vector of competition values for each taxon
$c = (c_1, c_2, \ldots c_x)$

For each taxon Y_j, there is an associated vector r_j of all stored metabolites
$r\_j = (r\_j_1, r\_j_2, \ldots r\_j_m)$

For each taxon Y_j there is a set s_j that gives the metabolites in r_j required by the taxon
$s\_j = \{s\_j_1, s\_j_2, \ldots s\_j_n\}$

For each taxon Y_j there is a set d_j that gives the metabolites in r_j excreted by the taxon
$d\_j = \{d\_j_1, d\_j_2, \ldots d\_j_q\}$

There is a single vector e for environmental metabolites, where entries in e give the amount of each metabolite in the environment
$e = (e_1, e_2, \ldots e_m)$

In addition, for each metabolite z, there is an associated matrix $CFbinary\_z$ (with dimensions of x by x) where the entry is 1 if the taxon in the given row has a cross-feeding relationship where it received metabolites from the taxon from the given column. All other entries are zero. This matrix is randomly generated in each model run using the process described in the main text.

The vector k_z contains the sum of each column in $CFbinary\_z$, which gives the number of cross-feeding donor relationships that each taxon has for metabolite z.

Dividing each entry in binary matrix $CFbinary\_z$ by the associated column sum in vector k_z yields the matrix $CFnorm\_z$, where each non-zero entry gives the fraction of excreted metabolites that are available for transfer from the taxon in the given column to the taxon in the given row.

Equations governing abundances and metabolites:

The model progresses through discrete time steps, where the vectors Y, r, and e are updated based on the prior values. The change to the abundance of taxon j through time is given by:

$$Y_j(t+1) = (Y_j(t) + min_{a \in s\_j}(r\_j_a(t) + h\_j_a(t))) * (1 - f) \qquad Eq.\ 1$$

Where $h\_j(t)$ is the vector giving the amount of each metabolite uptaken by taxon j from the environment in that time step:

$$\begin{aligned} h\_j(t) = (& \frac{c_j(Y_j(t) - r\_j_1(t))}{\sum_{k=1}^{x} c_k(Y_k(t) - r\_k_1(t))} * min(e_1(t) + i, \sum_{k=1}^{x} c_k(Y_k(t) - r\_k_1(t))), \\ & \frac{c_j(Y_j(t) - r\_j_2(t))}{\sum_{k=1}^{x} c_k(Y_k(t) - r\_k_2(t))} * min(e_2(t) + i, \sum_{k=1}^{x} c_k(Y_k(t) - r\_k_2(t))), ... \\ & \frac{c_j(Y_j(t) - r\_j_m(t))}{\sum_{k=1}^{x} c_k(Y_k(t) - r\_k_m(t))} * min(e_m(t) + i, \sum_{k=1}^{x} c_k(Y_k(t) - r\_k_m(t)))) \end{aligned} \qquad Eq.\ 2$$

Entries in h_j are zero if the metabolite is not required by the taxon.

Or, if Eq. 1 were described verbally,

The abundance at the next time step = (abundance at prior time step + minimum of (stored resources + resources uptaken from the environment)) * (1 – flushing)

The number of reproducing individuals from each taxon, given in the vector $g(t)$, is equal to the minimum of each taxon's stored metabolites plus metabolites uptaken from the environment:

$$\begin{aligned} g(t) = (& min_{a \in s\_1}(r\_1_a(t) + h\_1_a(t)), \\ & min_{a \in s\_2}(r\_2_a(t) + h\_2_a(t)), ... \\ & min_{a \in s\_x}(r\_x_a(t) + h\_x_a(t))) \end{aligned} \qquad Eq.\ 3$$

Multiplying the normalized cross-feeding matrix $CFnorm\_z$ by the reproduction vector $g(t)$ (which is equivalent to excretion, as excretion is coupled to reproduction) yields a vector $w\_z(t)$ where each entry is the quantity of metabolite z available to each taxon through cross-feeding:

$$w\_z(t) = CFnorm\_z * g(t) \qquad Eq.\ 4$$

At each time step, the sum of vector cf_z gives the total quantity of metabolite z exchanged through cross-feeding, with each entry corresponding to the quantity being acquired by each

taxon. The number of metabolites acquired by each taxon is the minimum of the number of metabolites available and the taxon's demand for metabolites:

$$cf\_z(t) \;=\; (\, min\,(\, w\_z_1(t)\,,\; Y_1(t) \;+\; 2\,g_1(t) \;-\; r\_1_z(t)\,)\;, \quad min\,(\, w\_z_2(t)\,,\; Y_2(t) \;+\; 2\,g_2(t) \;-\; r\_2_z(t)\,)\;, \ldots \quad min\,(\, w\_z_x(t)\,,\; Y_x(t) \;+\; 2\,g_x(t) \;-\; r\_x_z(t)\,)\;) \qquad \textit{Eq. 5}$$

The following equation governs the change in stored metabolites for taxon j over time:

$$r\_j\,(t+1) \;=(\,(\, r\_j_1(t) \;+\; h\_j_1(t) \;-\; g_1(t) \;+\; cf\_1_j(t)\,) \,*\, (1 \;-\; f)\,, \quad (\, r\_j_2(t) \;+\; h\_j_2(t) \;-\; g_2(t) \;+\; cf\_2_j(t)\,) \,*\, (1 \;-\; f)\;, \ldots \quad (\, r\_j_m(t) \;+\; h\_j_m(t) \;-\; g_m(t) \;+\; cf\_m_j(t)\,) \,*\, (1 \;-\; f)\;) \qquad \textit{Eq. 6}$$

Or, verbally,

The number of metabolites stored by a taxon equals the number of metabolites previously stored, plus the metabolites uptaken from the environment, minus the metabolites used for reproduction, plus the number of metabolites acquired from cross-feeding, with a fraction f lost to flushing.

The equations governing the invasive taxon's resource storage are the same as Eq. 6, but removing the cross-feeding term.

Finally, the amount of metabolite z in the environment can be written as:

$$e_z(t+1) \;=(\, e_z(t) \;+\; i \;-min\,(\, e_z(t) \;+\; i,\; \sum_{k=1}^{x} c_k(Y_k(t) \;-\; r\_k_z(t)\,) \;+\; \sum_{k=1}^{x} g_k(t) \,*\, \delta_k \;-\; \sum_{k=1}^{x} cf\_z_k\,) \,*\, (1-f)$$

$$\text{Where } \delta_k \;= \{1,\; \textit{if } z \in d\_k\,;\; 0,\; \textit{otherwise} \qquad \textit{Eq. 7}$$

Or, verbally,

The quantity of metabolites in the environment equals the previous quantity of metabolites in the environment, plus inputs, minus metabolites uptaken through competition, plus excreted metabolites but not those that were directed to other taxa through cross-feeding, and with a fraction f lost to flushing.
